# Syndromic Retinitis Pigmentosa: A Narrative Review

**DOI:** 10.3390/vision9010007

**Published:** 2025-01-20

**Authors:** Márta Janáky, Gábor Braunitzer

**Affiliations:** 1 Department of Ophthalmology, Szent-Györgyi Albert Medical School, University of Szeged, 6720 Szeged, Hungary; 2 Sztárai Institute, University of Tokaj, 3950 Sárospatak, Hungary; braunitzer.gabor@gmail.com

**Keywords:** inherited retinal dystrophies, syndromic retinitis pigmentosa, genetic mutations, systemic syndromes, multidisciplinary care

## Abstract

Retinitis pigmentosa (RP) encompasses inherited retinal dystrophies, appearing either as an isolated eye condition or as part of a broader systemic syndrome, known as syndromic RP. In these cases, RP includes systemic symptoms impacting other organs, complicating diagnosis and management. This review highlights key systemic syndromes linked with RP, such as Usher, Bardet–Biedl, and Alström syndromes, focusing on genetic mutations, inheritance, and clinical symptoms. These insights support clinicians in recognizing syndromic RP early. Ocular signs like nystagmus and congenital cataracts may indicate systemic disease, prompting genetic testing. Conversely, systemic symptoms may necessitate eye exams, even if vision symptoms are absent. Understanding the systemic aspects of these syndromes emphasizes the need for multidisciplinary collaboration among ophthalmologists, pediatricians, and other specialists to optimize patient care. The review also addresses emerging genetic therapies aimed at both visual and systemic symptoms, though more extensive studies are required to confirm their effectiveness. Overall, by detailing the genetic and clinical profiles of syndromic RP, this review seeks to aid healthcare professionals in diagnosing and managing these complex conditions more effectively, enhancing patient outcomes through timely, specialized intervention.

## 1. Introduction

Ocular manifestations are observed in various inherited diseases, often exacerbating the underlying systemic abnormalities. Advances in medical technology, along with a deeper understanding of the human genome and cellular anatomy, have led to significant progress in the diagnosis, classification, and, in some cases, treatment of these disorders. The two most well-known and frequently occurring forms of “syndromic retinitis pigmentosa” are Usher syndrome and Bardet–Biedl syndrome. However, many other inherited systemic diseases with retinal involvement are less familiar, both to ophthalmologists and pediatricians. Greater awareness of these associations is crucial for accurate diagnosis and understanding their clinical implications.

This review progresses through major categories of syndromic retinitis pigmentosa, beginning with ciliopathies such as Bardet–Biedl and Alström syndromes. It then addresses metabolic disorders, including Bassen–Kornzweig syndrome and peroxisomal diseases, followed by neurological conditions like Friedreich’s ataxia and Flynn–Aird syndrome, and mitochondrial diseases such as NARP syndrome and maternally inherited Leigh’s syndrome. Within each category, the review explores genetic mutations, inheritance patterns, and both systemic and ophthalmological manifestations, offering a thorough resource to support clinicians in their diagnostic and therapeutic approaches.

This review is a narrative review. To gather information, we conducted targeted searches across academic databases such as PubMed, Scopus, and Web of Science, focusing on peer-reviewed articles that address key aspects of syndromic retinitis pigmentosa and associated conditions. We also consulted online resources such as OMIM (Online Mendelian Inheritance in Man) to include established foundational knowledge.

## 2. Ciliopathies

Ciliopathies are a genetically heterogeneous group of disorders caused by mutations in genes whose products are localized to the cell’s cilium-centrosome complex [1,2,3].

### 2.1. Bardet–Biedl Syndrome (BBS, OMIM: 209900-619471)

Also known as Laurence–Moon–Bardet–Biedl syndrome (LMBBS), adipogenital retinitis pigmentosa syndrome (Laurence–Moon–Biedl syndrome). Although BBS and LMBBS are considered “independent” diseases, due to their overlapping systemic features and inheritance patterns, they are often regarded as the same condition. Mutations in the *BBS1* gene have been reported more frequently [4,5].

Bardet–Biedl Syndrome encompasses a genetically and clinically heterogeneous group of disorders, with at least 22 identified subtypes, each associated with mutations in distinct genes, including BBS1 (OMIM #209900), BBS2 (OMIM #615981), BBS3 (OMIM #600151), BBS4 (OMIM #615982), BBS5 (OMIM #615983), BBS6 (OMIM #605231), BBS7 (OMIM #615984), BBS8 (OMIM #615985), BBS9 (OMIM #615986), BBS10 (OMIM #615987), BBS11 (OMIM #615988), BBS12 (OMIM #615989), BBS13 (OMIM #615990), BBS14 (OMIM #615991), BBS15 (OMIM #615992), BBS16 (OMIM #615993), BBS17 (OMIM #615994), BBS18 (OMIM #615995), BBS19 (OMIM #615996), BBS20 (OMIM #619471), BBS21 (OMIM #617406), and BBS22 (OMIM #617119), with cytogenetic locations ranging from 1p35.2 to 22q12.3, reflecting the complex molecular pathways underlying its characteristic clinical features such as retinal dystrophy, polydactyly, obesity, and renal abnormalities.

The underlying cause of the developmental and retinal abnormalities in these disorders is ciliary dysgenesis (ciliopathy) [6]. Evidence suggests that BBS involves disruptions in the melanocortin-4 receptor (MC4R) pathway, a critical mechanism for regulating energy homeostasis and body weight. Research demonstrates that setmelanotide, an MC4R agonist, effectively counteracts deficiencies in this pathway, leading to significant improvements in weight and hunger management in individuals with BBS [7]. Additionally, disruptions in the BBSome complex impair MC4R trafficking and localization in cilia, exacerbating obesity and affecting energy regulation [8]. These impairments highlight the critical role of BBS proteins in maintaining functional ciliary signaling [9].

Moreover, the BBSome complex functions as an adapter for G protein-coupled receptors (GPCRs), including MC4R, ensuring proper intra-cellular trafficking and signaling, thereby maintaining energy balance [10]. Long-term clinical studies confirm that prolonged use of MC4R agonists like setmelanotide is effective in reducing BMI and managing hyperphagia in BBS patients, reinforcing its therapeutic relevance [11].

BBS is associated with triallelic digenic mutations in at least 11 genes, with an autosomal recessive inheritance pattern [12]. Diallelic trigenic inheritance is a complex genetic inheritance pattern involving three distinct genes, each contributing two alleles (one from each parent in diploid organisms). The trait or condition arises from the combined effects of specific allele combinations across all three genes. Unlike simpler inheritance patterns, where a single gene or two genes determine a phenotype, trigenic inheritance requires interactions at three loci to manifest the trait. In this mode of inheritance, the phenotype is influenced by either additive or interactive effects of the alleles. Additive effects occur when each allele independently contributes to the trait, while interactive effects involve specific combinations of alleles across the three genes working together to produce a significant outcome. Importantly, the trait or disorder may only appear if a certain threshold of genetic contributions is met, meaning not all individuals carrying risk alleles at these genes will exhibit the condition.

Systemic symptoms of this syndrome typically present in childhood and include obesity, microcephaly, intellectual disabilities (including mental retardation), and mild psychomotor delay. Additional physical findings may include post-axial polydactyly and syndactyly. In males, common abnormalities include hypogonadism, micropenis, bifid scrotum, and cryptorchidism, while females may exhibit vaginal atresia, a double uterus, primary amenorrhea, and hypospadias. Renal abnormalities are also frequently observed.

Less common systemic signs and symptoms may include speech difficulties, deafness, seizures, epilepsy, cerebellar ataxia, cardiac disease, diabetes, progeria, premature aging, and ichthyosis [13,14,15].

Ophthalmological signs commonly observed in individuals with BBS include external ophthalmoplegia and retinal findings such as rod–cone dystrophy or cone–rod dystrophy. These retinal changes are often consistent with a diagnosis of retinitis pigmentosa [16,17,18].

### 2.2. Alström Syndrome (AS, OMIM: 203800)

Also known as Alström syndrome, retinitis pigmentosa–deafness–obesity–diabetes mellitus, Hallgren–Alström syndrome, retino-otodiabetic syndrome, or Alström–Hallgren syndrome. The syndrome was first described by Alström in 1959 [19]. While the symptoms resemble those of Bardet–Biedl syndrome (BBS), Alström syndrome is distinguished by the absence of polydactyly, hypogenitalism, and intellectual disabilities. Diagnostic criteria include hearing loss, obesity, diabetes mellitus (DM), acanthosis nigricans, and retinitis pigmentosa (RP). The syndrome is caused by mutations in the *ALMS1* gene, located on chromosome 2 (2p13) [20,21]. Inheritance is autosomal recessive (autosomal recessive).

Systemic symptoms associated with Alström syndrome commonly include hearing loss and deafness. Cardiovascular conditions such as cardiomyopathy and cardiomegaly are frequently observed. Obesity and type 2 diabetes mellitus (T2DM, characterized by insulin resistance) are also prevalent. Less commonly reported systemic findings include acanthosis nigricans, gout, hypothyroidism, kyphoscoliosis, and chronic nephropathy [22,23,24,25,26].

Ophthalmological signs often seen in Alström syndrome include nystagmus, photophobia, and divergent strabismus. Retinal changes such as cone–rod dystrophy contribute to progressive vision loss and eventual blindness [27].

### 2.3. Joubert Syndrome (JBTS, OMIM: 213300)

Described by Joubert in 1966 [28], JBTS is a phenotypically heterogeneous syndrome that includes various central nervous system (CNS) developmental abnormalities, such as the so-called “molar tooth sign”, cerebellar vermis hypoplasia, and cerebral cortex defects [29,30]. There are overlaps with Senior–Loken syndrome, as *NPHP1* is a causative factor in about 2% of JBTS4 cases. Another causative gene, *AHI1*, has been recently discovered in the JBTS3 form. In addition, two other loci, JBTS1 and JBTS2, have been identified [31]. The inheritance is autosomal recessive, but there are also X-linked recessive (XL-R) forms [32].

Systemic symptoms associated with this condition include generalized motor developmental delay, hypotonia, ataxia, intellectual disability, episodic hyperpnea and apnea, and characteristic facial features such as widely spaced eyes and multicystic kidney disease [33,34,35].

Ophthalmological signs commonly observed include ocular motor apraxia, abnormal eye movements, nystagmus, ptosis, retinal coloboma, and retinal dystrophy [36,37,38].

### 2.4. Senior–Løken Syndrome (SLS, OMIM: 266900)

Also referred to as Løken–Senior syndrome, renal–retinal syndrome, oculo-renal syndrome, juvenile nephronophthisis with Leber amaurosis, renal dysplasia, or retinal aplasia.

First described in 1961 [39], the syndrome involves mutations in at least 10 or more genes (such as *NPHP1* and *NPHP3-5*) that encode nephrocystins, which disrupt the structure and function of cilia cells [40,41]. The most common form, Senior–Løken syndrome type 1 (SLSN1), results from a homozygous mutation in the *NPHP1* gene on chromosome 2q13. It follows an autosomal recessive inheritance pattern.

Nephronophthisis can also appear in various syndromes that affect multiple body systems, known as nephronophthisis-associated ciliopathies. In Senior–Løken syndrome, this condition is combined with retinal degeneration, which involves the deterioration of the retina’s light-sensitive tissue [39,42,43].

Systemic symptoms include juvenile nephronophthisis, polyuria, polydipsia, bone malformations, hearing impairments, intellectual disability, and vascular narrowing [44].

Ophthalmological signs include ultra-low vision or complete blindness (absence of light perception) at birth or severe, progressive retinitis pigmentosa, which may sometimes be diagnosed as Leber’s congenital amaurosis (LCA) or as a milder form of retinitis pigmentosa diagnosed later in life [45].

### 2.5. Jeune Syndrome (OMIM: 208500)

Also known as asphyxiating thoracic dystrophy (ATD) or asphyxiating thoracic dysmorphy, this rare genetic disorder affects the development of cartilage and bones in children. Causative genes identified include *IFT80*, *DYNC2H1*, *WDR19*, *IFT140*, and *TTCZ1B*. Exome sequencing has shown that mutations in *DYNC2H1* are a common cause of the syndrome. The inheritance pattern is autosomal recessive [46].

Systemic symptoms include thoracic hypoplasia, breathing difficulties, brachydactyly, occasional polydactyly, kyphoscoliosis, scoliosis, chronic nephritis, and heart problems, which typically develop in later stages [46,47].

Ophthalmological signs include chorioretinal dystrophy, nyctalopia, significant peripheral visual field reduction [48], myopia, strabismus, astigmatism, lens opacification, iris atrophy, and colobomas of the eyelid or retina [49].

### 2.6. Kearns–Sayre Disease (OMIM: 530000)

Also referred to as Kearns-Sayre mitochondrial ciliopathy or oculocranio-somatic syndrome [50,51,52,53], this syndrome was first described in the mid-20th century [54,55]. It is most often caused by sporadic deletions in mitochondrial DNA, which can result from point mutations within the mtDNA (including tRNA genes) or in nuclear genes involved in mitochondrial DNA maintenance, such as *RRM2B*. The inheritance pattern is mitochondrial [56] or autosomal recessive [57].

Systemic symptoms include sensorineural deafness, cerebellar ataxia, dysarthria, bilateral facial weakness, skeletal muscle myopathy, intellectual disability, gastrointestinal disorders, cardiomyopathy, cardiac conduction defects, heart block, endocrine issues, delayed puberty, and hypoparathyroidism [58,59].

Ophthalmological signs include progressive external ophthalmoplegia, ptosis, night blindness, and pigmentary retinopathy, often presenting as salt-and-pepper retinopathy with diffuse stippled areas of retinal pigment epithelium hypopigmentation and peripapillary epithelial atrophy [50,60].

### 2.7. Usher Syndrome (OMIM: 276900)

Also known as Usher–Hallgren syndrome, retinitis pigmentosa–dysacusis syndrome, or dystrophia–dysacusis syndrome, this is the most common syndromic form in which retinitis pigmentosa is associated with neurosensory deafness [61]. Approximately 14% of all RP cases are in fact Usher syndrome. Ten genes have been identified as causative for Usher syndrome, with six more genes associated with the disease [62,63,64]. These genes encode a range of proteins that interact to form the “Usher interactome”, a dynamic protein network crucial to the syndrome. Usher syndrome is an autosomal recessive ciliopathy [65], characterized by sensorineural hearing loss, retinitis pigmentosa, and, in some cases, vestibular dysfunction. The syndrome manifests in three clinical types, depending on the severity and age of onset of symptoms.

Type 1 Usher syndrome is caused by mutations in the *CDH23*, *MYO7A*, *PCDH15*, *USH1C*, and *USH1G* genes [66,67]. Systemic symptoms of type 1 include profound congenital hearing loss or deafness, severe balance issues, vestibular ataxia, and difficulty walking before 18 months of age. In some instances, mental health issues, including signs of schizophrenia, may develop. Ophthalmological symptoms typically begin before the age of 10, starting with night blindness due to retinal pigment epithelium (RPE) and rod dysfunction. Over the following decades, vision progressively worsens, leading to severe vision loss. Additional ocular symptoms may include nystagmus, keratoconus, and optic atrophy.

In type 2 Usher syndrome, mutations in the *USH2A*, *GPR98*, and *DFNB31* genes are responsible [68,69,70,71]. The systemic symptoms include moderate to severe hearing loss present at birth, with normal balance. As the condition progresses, hearing impairment worsens, but individuals can generally communicate verbally and benefit from hearing aids. Some patients may experience issues with speech articulation. Ophthalmological signs, particularly retinitis pigmentosa, usually emerge in late adolescence.

Type 3 Usher syndrome is caused by mutations in the *CLRN1* gene, which encodes the clarin protein, a crucial component for the development of inner ear and retinal cells [72,73]. The exact mechanisms of its action are not fully understood. In type 3, systemic symptoms include normal hearing at birth, followed by progressive hearing loss during adolescence. Ophthalmological symptoms, such as night blindness, also typically begin in adolescence. Blind spots start to appear in the late teens to early twenties, and legal blindness often occurs by midlife. Note that legal blindness is not a medical term, rather a category used by government agencies to determine those who are eligible to receive disability benefits. The precise definition may vary depending on the jurisdiction. In the United States, for instance, a person is considered legally blind if he/she has central visual acuity of 20/200 or worse in the better-seeing eye with the best correction (using glasses or contact lenses) at a distance or if he/she has visual field restriction where the widest diameter is 20 degrees or less in the better-seeing eye [74].

The identification of the genetic alterations associated with Usher syndrome is important for prognosis.

## 3. Metabolic Diseases with Retinal Dystrophy

The cell membrane (plasma membrane) is primarily composed of lipid molecules, such as fatty-acid-based lipids and proteins. Defects in gene function affecting this membrane can result in various metabolic diseases.

### 3.1. Lipid Abnormalities

#### 3.1.1. Bassen–Kornzweig Syndrome (OMIM: 200100)

Also referred to as abetalipoproteinemia, this condition was first described by Bassen and Kornzweig in 1950 [75] and is caused by pathogenic variants in the *MTTP* gene, which encodes the microsomal triglyceride transfer protein [76]. The *MTTP* gene produces the large subunit of the heterodimeric microsomal triglyceride transfer protein (MTP), with protein disulfide isomerase (PDI) forming the second subunit. This protein complex is crucial for the assembly of lipoproteins. Mutations in *MTTP* lead to abetalipoproteinemia, a condition that disrupts fat and vitamin absorption [75,76,77,78]. The inheritance pattern of the disease is autosomal recessive.

Systemic symptoms include early-onset features such as diarrhea, vomiting, steatorrhea, delayed growth, and impaired bone development, typically manifesting in the first year of life or early childhood. In cases of later disease onset, additional neurological symptoms may emerge, including balance difficulties, muscle weakness, progressive ataxia, peripheral neuropathy, slurred speech, cardiomyopathy, and liver complications [79,80,81,82]

Ophthalmological signs include difficulties with vision in low-light conditions, constriction of the visual field, and a form of pigmentary retinopathy resembling retinitis punctata albescens, with angioid streaks. Other ocular findings may include nystagmus, anisocoria, ptosis, eye muscle paralysis, ophthalmoplegia, and cataracts [83,84].

#### 3.1.2. Hooft Disease (OMIM: 236300)

Also referred to as Hooft’s syndrome, hypolipidemia syndrome, or hypolipidemia-tryptophan abnormality, this condition was first described in 1962 by Hooft and colleagues [85]. The most common cause is a mutation in the *CLN3* gene, with symptoms typically manifesting at a young age. The inheritance pattern is autosomal recessive.

Systemic symptoms include erythematosquamous eruptions, red rashes, opaque leukonychia, intellectual disability, cognitive impairment, delayed growth, and acute lower gastrointestinal bleeding [86].

Ophthalmological manifestations include rod–cone dystrophy, with more severe involvement of the rod photoreceptors compared to the cone photoreceptors [87]. Rod–cone dystrophy is an umbrella term for a group of inherited retinal disorders marked by the progressive degeneration of rod and cone photoreceptors in the retina. The condition typically starts with night blindness and peripheral vision loss due to rod dysfunction, eventually advancing to central vision impairment as cone photoreceptors deteriorate. This genetically diverse disorder is associated with mutations in various genes, such as *ABCA4*, *RPGR*, and *GUCY2D*. Although primarily non-syndromic, rod–cone dystrophy can also occur as part of syndromic conditions [88,89].

#### 3.1.3. Familial Combined Hypolipidemia (OMIM: 144250)

Also referred to as hypo-abetalipoproteinemia, this familial combined hypolipidemia is a disorder of Mendelian inheritance marked by significant reductions in all three major lipid fractions (LDL-C, HDL-C, triglycerides), resulting from mutations that inactivate the angiopoietin-like 3 (*ANGPTL3*) gene [90]. The condition follows an autosomal dominant inheritance pattern [91,92,93].

The systemic and ophthalmological symptoms mirror those observed in Hooft disease.

### 3.2. Peroxisomal Diseases

Peroxisomes are membrane-bound intra-cellular organelles that play a crucial role in catalyzing various processes in cellular metabolism and biosynthesis, including the beta-oxidation of very long-chain fatty acids (VLCFAs). Metabolic disorders arise when there is a disruption in peroxisome biogenesis or in one of their metabolic functions [94,95].

Peroxisomal disorders represent a heterogeneous group of inherited metabolic conditions marked by dysfunctional lipid metabolism, which arise when peroxisomes are either absent or fail to function correctly in the body [94]. One prominent example is Zellweger spectrum disease (ZSD), also known as cerebro-hepato-renal syndrome. This disorder occurs due to the absence or reduction of functional peroxisomes, which are essential for processes like the beta-oxidation of very long-chain fatty acids. Mutations in at least 13 different *PEX* genes can lead to the symptoms observed in ZSD.

The term Zellweger spectrum disease encompasses a range of conditions, including Refsum disease (both the adult and infantile forms), rhizomelic chondrodysplasia punctata, X-linked adrenoleukodystrophy (ALD), Zellweger syndrome (ZS), and neonatal adrenoleukodystrophy (NALD). These are discussed below in more detail. There is considerable overlap in the clinical features of Zellweger syndrome, neonatal adrenoleukodystrophy, and infantile Refsum disease, which are collectively referred to as the Zellweger spectrum disorders.

Zellweger syndrome is the most severe form within the spectrum of peroxisome biogenesis disorders (PBDs). In contrast, neonatal adrenoleukodystrophy and infantile Refsum disease, both milder forms of cerebro-hepato-renal syndrome, represent the less severe manifestations of these disorders [96].

#### 3.2.1. Classic Refsum Disease (OMIM: 266500)

The adult form of Refsum disease, also known as “Classic” Refsum disease [97], is a disorder characterized by the accumulation of phytanic acid in the body. This condition arises due to mutations in the gene responsible for producing phytanoyl-CoA hydroxylase (*PAHX* or *PHYH*) or the gene that encodes peroxin-7 (*PEX7*). Phytanoyl-CoA hydroxylase, produced by the *PHYH* and *PAHX* genes, is crucial for the alpha-oxidation of branched-chain fatty acids. Mutations in these genes result in significantly elevated levels of phytanic acid in the body [98,99,100,101,102].

The disease follows an autosomal recessive pattern of inheritance. Systemic symptoms include anosmia, deafness, and various skin abnormalities [103]. The ophthalmological sign is retinitis pigmentosa [104,105].

#### 3.2.2. Zellweger Syndrome (OMIM: 214100)

Also known as cerebro-hepato-renal syndrome, this syndrome results from the absence or reduction of functional peroxisomes, which are essential for the beta-oxidation of very long-chain fatty acids. Mutations in at least 13 *PEX* genes can lead to the condition, with the most common mutations found in the *PEX1* and *PEX6* genes. The inheritance pattern of the syndrome is autosomal recessive [106,107].

Systemic symptoms include reduced spontaneous movement, hypotonia, difficulties with sucking or swallowing, cognitive deficits, and possible seizures in the neonatal and infantile forms. Systemic signs include liver dysfunction, jaundice, kidney and gastrointestinal issues such as bleeding, and characteristic facial dysmorphisms. Additional features include hearing impairment [108].

Ophthalmological signs include retinal degeneration characterized by leopard spot pigmentation, retinal arteriolar attenuation, optic atrophy, and, in some cases, corneal clouding, glaucoma, or cataracts [109,110].

#### 3.2.3. Neonatal Adrenoleukodystrophy (NALD, OMIM: 601539)

The inheritance pattern of this condition is either autosomal recessive or X-linked recessive [111,112,113]. The phenotypic spectrum of NALD/IRD peroxisome biogenesis disorders can be caused by mutation in members of the peroxin (*PEX*) gene family. The *PEX* genes encode proteins essential for the assembly of functional peroxisomes [114].

Systemic symptoms include psychomotor delay, deafness, seizures, and failure to thrive, while systemic signs include muscle hypotonia [112,115].

Ophthalmological signs include blindness, typically developing within the first few weeks of life. Retinopathic leopard spots can aid in diagnosis [109].

#### 3.2.4. Juvenile Adrenoleukodystrophy (ALD, OMIM: 300100)

This condition is caused by a mutation in the *ABCD1* gene, which is located on the X chromosome. The inheritance pattern is X-linked recessive.

Systemic signs include progressive neurological deterioration due to demyelination of the cerebral white matter. Systemic symptoms include behavioral problems, poor memory, difficulty with reading, writing, and understanding speech. Additional symptoms may include headaches, seizures, loss of muscle control, and progressive dementia.

The condition has only one ophthalmological sign, which is blindness resulting from optic nerve demyelination [109].

#### 3.2.5. Infantile Refsum Disease (IRD, OMIM: 266510)

This condition is caused by mutations in the *PEX1*, *PEX2*, *PEX26*, *PHYH*, or *PEX7* genes [100]. Infantile Refsum disease, which is the least severe form of Zellweger spectrum disorder, remains a fatal disease [116,117]. The inheritance pattern is autosomal recessive.

Systemic signs include ataxia, polyneuropathy, hypotonia, mild facial dysmorphism, and cardiomyopathy. Systemic symptoms include anosmia, sensorineural hearing loss, growth retardation, and, rarely, epidermal abnormalities [103].

Ophthalmological signs typically begin in infancy with retinitis pigmentosa [109,116]. This condition leads to blindness.

#### 3.2.6. Neuronal Ceroid Lipofuscinosis (OMIM: 256730)

Also known as amaurotic idiocy, this condition involves a protein encoded by the *CLN3* gene, which is found in lysosomes and synapses [118,119]. The inheritance pattern is predominantly autosomal recessive, but there are autosomal dominant forms. Four distinct forms are differentiated between, according to the age of onset:

1. The infantile form (Santavouri–Haltia disease) presents with symptoms around 6 to 8 months of age [120].
2. The late infantile form (Jansky–Bielschowsky disease) starts between 2 and 4 years of age. The first signs are ataxia and mental retardation, and the disease typically leads to death between 8 and 12 years of age [121].
3. The juvenile form (Batten disease, Spielmeyer–Vogt disease) appears between 5 and 8 years of age, with progressive vision loss, seizures, and mental retardation. This is the most frequent form [122].
4. The adult form (Kufs disease) begins before 30 to 40 years of age and is considered the milder form of the disease [123].

Systemic symptoms include mental retardation, seizures, ataxia, and acute spasms [124].

Ophthalmological signs begin with macular involvement, characterized by cherry-red spots, followed by progressive degeneration spreading to the peripheral retina, leading to retinal dystrophy and blindness. The disease typically results in death between 10 and 15 years of age [125].

#### 3.2.7. HARP Syndrome (OMIM: 234200)

The syndrome—involving hypoprebetalipoproteinemia, acanthocytosis, retinitis pigmentosa, and pallidal degeneration—is a specific form of pantothenate kinase-associated neurodegeneration (PKAN) [126,127], and is now considered an NBIA (neurodegeneration with brain iron accumulation) disorder [128,129,130,131].

PKAN is caused by the loss of function of the enzyme PANK2 due to mutations in the *PANK2* gene, which encodes pantothenate kinase 2, located on chromosome 20p13 [126,127,132,133].

The syndrome was also known as Hallervorden–Spatz syndrome, but the name has been abandoned due to the eponymous doctors’ participation in Nazi atrocities [134].

Patients have decreased or absent pre-beta lipoproteins, which are composed of very low-density lipoproteins. This is considered a spectrum disease. The neurological symptoms arise from iron deposits in the basal ganglia of the brain, caused by the enzyme defect [135,136]. The inheritance pattern is autosomal recessive.

Systemic signs include neurodegeneration, acanthocytosis, and pallidal degeneration. Systemic symptoms include progressive extrapyramidal dysfunction, dystonia, dysarthria, rigidity, dementia, and parkinsonism [131,137,138].

Ophthalmological signs include retinitis pigmentosa, optic atrophy, abnormal pupillary reactions, color vision deficiency, constriction of the visual field, and flecked-type retinopathy with bull’s eye maculopathy. Ophthalmological symptoms include night blindness and, eventually, total blindness [139,140].

### 3.3. Mucopolysaccharidoses (MPSs)

Mucopolysaccharidoses (MPS) are a group of disorders caused by inherited defects in lysosomal enzymes, leading to the widespread intra- and extra-cellular accumulation of glycosaminoglycans. These disorders are subdivided based on the specific enzyme defect and their systemic manifestations [141].

The most well-known forms of these metabolic diseases include Hurler, Hunter, Sanfilippo, and Scheie syndromes, while lesser-known forms include Morquio, Maroteaux-Lamy, Sly, and Natowicz syndromes. Inheritance is generally autosomal recessive, with the exception of Hunter syndrome, which follows an X-linked recessive inheritance pattern.

Only the Hurler, Hunter, and Sanfilippo syndromes are associated with pigmentary retinopathy [142,143,144,145,146].

#### 3.3.1. Hurler Syndrome (OMIM: 607014)

Also known as gargoylism, this condition is a lysosomal storage disease caused by defects in the enzyme alpha-L-iduronidase, encoded by the *IDUA* gene on chromosome 4p16. It is the most severe form of mucopolysaccharidosis, classified as MPS I-H. The genetic defect results in the accumulation of glycosaminoglycans (GAGs) both inside and outside cells within lysosomes [147].

The condition follows an autosomal recessive inheritance pattern. Systemic symptoms usually appear in early childhood and include characteristic facial and skeletal changes. Affected individuals often have an abnormally large head, a prominent forehead, a flattened nose, and swollen lips. Growth typically ceases around the age of two, and intellectual disability, as well as gastrointestinal, cardiac, and kidney issues, are common. The disease is progressive, and life expectancy is generally around 10 years of age [148].

Ophthalmological signs include exophthalmos, retinal dystrophy, and early-onset corneal clouding. Glaucoma may also occur, though this is rare [142,143,144,146].

#### 3.3.2. Hunter Syndrome (OMIM: 309900)

Described in 1917 [149], Hunter syndrome follows an X-linked recessive inheritance [150,151]. It is caused by mutation in the gene encoding iduronate 2-sulfatase (*IDS*) on chromosome Xq28. Systemic signs include noticeable skeletal changes and enlargement of the liver and spleen (hepatosplenomegaly). Systemic symptoms include intellectual disability.

Ophthalmological signs include retinal dystrophy, often characterized by abnormal pigmentation of the fundus. Ophthalmological symptoms include night blindness.

#### 3.3.3. Sanfilippo Syndrome (OMIM: 252900)

This is a syndrome with autosomal recessive inheritance. The syndrome is caused by homozygous or compound heterozygous mutation in the gene encoding N-sulfoglucosamine sulfohydrolase (*SGSH*) on chromosome 17q25. The clinical picture is dominated by mental retardation and skeletal changes; additionally, the constriction of the visual field caused by retinal dystrophy further aggravates the patients’ condition. [152,153].

### 3.4. Other Rare Metabolic Diseases with Retinal Involvement

#### 3.4.1. Methylmalonic Aciduria with Homocystinuria (MMACHC, OMIM: 277400)

MMACHC is caused by genetic defects in enzymes responsible for metabolizing vitamin B12. Mutations in the *MMACHC* gene (1p36.3) cause the cblC subtype, while mutations in the *MMADHC* gene (2q23.2) cause the cblD subtype. Mutations in the *LMBRD1* gene (6q13) lead to the cblF subtype, and mutations in the *ABCD4* gene result in the cblJ subtype [154,155]. The condition is inherited in an autosomal recessive manner.

Systemic signs include microcephaly, mild facial dysmorphism, hydrocephalus, white matter abnormalities, basal ganglia lesions, megaloblastic anemia (characterized by pallor), cardiac malformations, skin rashes, and hypotonia. Systemic symptoms include developmental delays, dementia, ataxia, behavioral problems, learning difficulties, fatigue, anorexia, stomatitis, psychosis, and feeding difficulties. Those who become symptomatic beyond infancy may present with ataxia, dementia, psychosis, or feeding difficulties [156,157].

Ophthalmological signs include salt-and-pepper retinopathy, vascular attenuation, and macular atrophy [158,159].

#### 3.4.2. Cystinosis (OMIM: 219800)

Cystinosis is a rare multisystem genetic disorder and a lysosomal storage disease in which cystine accumulates in organs and tissues throughout the body. The condition is caused by mutations in the *CTNS* gene, which encodes a protein called cystinosin. This protein is involved in the lysosomal transmembrane transport of cystine. The characteristic crystal deposits seen in the disease result from the accumulation of cystine in various parts of the body. The disorder follows an autosomal recessive inheritance pattern [160,161,162,163].

Systemic signs include short stature, hypothyroidism, diabetes mellitus, and renal failure [164].

Ophthalmological signs include retinal alterations, the presence of typical crystal deposits in the cornea, and pigmentary retinopathy. Ophthalmological symptoms include photophobia [165,166].

## 4. Neurological Disorders with Retinal Dystrophy

### 4.1. Hereditary Ataxias

Among the hereditary ataxias, two main types are distinguished where generalized tapetoretinal degeneration may occur. These include Friedreich’s ataxia, which follows an autosomal recessive inheritance pattern, and Marie’s ataxia, which is inherited in an autosomal dominant manner.

#### 4.1.1. Friedreich Ataxia (OMIM: 229300)

Also known as Friedreich’s hereditary spinocerebellar ataxia, this condition is caused by mutations in the *FRDA* gene (frataxin) located on chromosome 9 (9q13-q21) or the *FRDA2* gene (9p23-p11). These mutations lead to cerebellar abnormalities, including degeneration of the posterior column of the spinal cord, the spinocerebellar and corticospinal tracts, and segmental demyelination [167,168]. The inheritance pattern is autosomal recessive.

Systemic signs include skeletal abnormalities such as pes equinovarus, pes cavus, kyphoscoliosis, and short stature. Other signs include clumsy hands and legs, respiratory incoordination, speech incoordination, and cardiomyopathy in later stages. Systemic symptoms typically appear between the ages of 6 and 25 and include balance disturbances, ataxia, acute muscle spasms, and sometimes hearing loss. There is no associated intellectual disability [169,170,171].

Ophthalmological signs include nystagmus, optic atrophy, and retinal dystrophy [172,173,174,175].

#### 4.1.2. Marie’s Ataxia (Olivopontocerebellar Atrophy, OMIM: 164400)

This is an autosomal dominant variant of Friedreich’s ataxia [176]. It is caused by an expanded (CAG)n trinucleotide repeat in the ataxin-1 gene (*ATXN1*) on chromosome 6p22. Autosomal dominant cerebellar ataxia (ADCA1) is a progressive movement disorder caused by atrophy of nerve cells in the cerebellum. There is some uncertainty in differentiating it from other cerebellar ataxias with autosomal dominant inheritance. Various types of autosomal dominant cerebellar ataxia have been identified, sometimes collectively referred to as “other dominant cerebellar ataxia”, based on different gene defects such as ADCA1, SYNE1, or SCA7 [177,178,179].

Three distinct types are differentiated according to their phenotypic and genotypic characteristics. Among them, type II (SCA7) also presents with retinal disease [177,180,181,182].

Systemic symptoms, which may appear in early life or later, include progressive spastic paraplegia, dysarthria, dystonic choreoathetosis, and uncontrolled acute movements in some muscles of the body, lasting from one minute to several hours [183,184,185].

Ophthalmological signs include nystagmus, optic atrophy, retinal dystrophy, and granular retinal alterations that typically begin in the macula and progressively spread to the entire retina [186].

### 4.2. Other Neurological Diseases

#### 4.2.1. Flynn–Aird Syndrome (OMIM: 136300)

This is a rare inherited neurological disease with childhood onset, caused by an enzyme defect, although its genetic background is not fully understood. Several symptoms overlap with known neurological disorders such as Werner syndrome, Refsum syndrome, and Cockayne syndrome, suggesting possible similar origins. However, these syndromes are recessively inherited, while the condition described by P. Flynn and Robert B. Aird follows an autosomal dominant inheritance pattern [187]. Due to skin changes, including skin atrophy and alopecia, the condition is classified as a neurodermal syndrome.

Systemic signs include skeletal abnormalities such as cystic bone alterations and deformities, endocrinological problems, dental problems, skin atrophy, and alopecia. Systemic symptoms, which typically appear between the ages of 10 and 20 and develop progressively, include dementia, aphasia, ataxia, muscular and peripheral neuropathy, epilepsy, and progressive binocular hearing loss.

Ophthalmological signs, which typically progress between 20 and 30 years of age, include retinitis pigmentosa, myopia, and cataracts.

#### 4.2.2. Sjögren–Larson Syndrome (SLS, OMIM: 270200)

This syndrome is caused by a deficiency of fatty aldehyde dehydrogenase, resulting from mutations in the *ALDH3A2* gene. The enzyme deficiency leads to impairments in essential bodily functions, particularly affecting the brain [188,189,190,191].

Systemic signs include ichthyosis, initially observed on the neck and lower parts of the arms. Systemic symptoms include developmental delays, intellectual disability, spasm attacks, and speech difficulties [191].

Ophthalmological signs include retinal pigment degeneration, characterized by glittering white spots [192,193].

## 5. Mitochondrial Diseases

### 5.1. NARP Syndrome (OMIM: 551500)

This syndrome is characterized by neuropathy, ataxia, and retinitis pigmentosa, which gives the condition its name. The disorder results from abnormal mitochondrial energy production, caused by specific mutations in the *MT-ATP6* gene. The most common mutations are m.8993 T>G and m.8993 T>C, leading to deficits in ATP production [194,195,196,197]. The inheritance pattern is autosomal recessive [196,197].

Systemic signs include cardiomyopathy and sensory peripheral neuropathy. Systemic symptoms, which typically appear in early childhood with intermittent progression and periods of stability, include breathing difficulties, spasm attacks, migraines, slow development, muscle weakness, ataxia, hearing loss, anxiety, and sleep disturbances [198]. Unlike other mitochondrial diseases, this syndrome does not typically cause lactic acidosis.

Ophthalmological signs include abnormal pupillary reflexes, nystagmus, salt-and-pepper retinopathy, macular degeneration, and ophthalmoplegia [199].

A more severe form of the condition can occur if over 90% of the mitochondrial DNA is affected by the mutation [200].

### 5.2. MILS Disease (Maternally Inherited Leigh’s Syndrome, OMIM: 500017)

This disease is caused by a mutation in mitochondrial DNA (mtDNA) [201,202,203]. The causative genes include is caused by mutation in several mitochondrial genes, including *MTTV*, *MTTK*, *MTTW*, *MTTL1*, and *MTATP6*.

Systemic signs include cardiomyopathy, lactic acidosis, and developmental abnormalities of the central nervous system. Systemic symptoms, which typically appear in infancy or early childhood, include encephalopathy, spasm attacks, and breathing difficulties [201,204].

Ophthalmological signs, similar to those seen in NARP syndrome, include macular degeneration and ophthalmoplegia [205].

### 5.3. Myotonic Dystrophy (OMIM: 160900)

Myotonic dystrophy (DM) is an inherited multisystem disorder that primarily causes progressive muscle loss, weakness, and myotonia. It can also affect various other parts of the body, including the heart, lungs, and eyes [206,207]. The condition follows an autosomal dominant inheritance pattern.

DM is classified into two types. Type 1 DM (DM1), also known as Steinert disease, is caused by an abnormally expanded section of a gene on chromosome 19 (*DMPK*), located near the regulatory region of another gene, *SIX5* [207]. Systemic symptoms include distal muscle weakness, myotonia, and muscle degeneration [208,209,210]. Ophthalmological signs include ptosis, cataracts, retinal pigment epithelium (RPE) dystrophy, patterned macular degeneration with yellow plaques, retinal vascular abnormalities, and epiretinal membrane [211,212,213,214,215,216].

Type 2 DM (DM2), recognized in 1994 as a milder form of DM1, is caused by an abnormally expanded section in the ZNF9 gene on chromosome 3. DM2 was initially known as proximal myotonic myopathy (PROMM), a term still in use but less common than DM2 [217,218]. Systemic symptoms include myotonia, distal weakness, and dystrophic muscle degeneration. Ophthalmological signs include cataracts, retinal pigment changes (retinal degeneration), RPE dystrophy, and glaucomatous optic neuropathy [216,219].

## 6. Dysmorphic Syndromes

### 6.1. Cohen Syndrome (OMIM: 216550)

One of the causative genes associated with this syndrome is *COH1*, which encodes a protein involved in vesicular trafficking. It is linked to variant mutations in the *VPS13B gene* [220,221,222]. The inheritance pattern is autosomal recessive.

Systemic signs include distinctive facial dysmorphism, such as prominent upper incisors, short stature, long and narrow hands, neutropenia, and hypotonia. Systemic symptoms include non-progressive intellectual disability or mental retardation [223].

Ophthalmological signs include fundus pigmentation and bull’s eye maculopathy. Ophthalmological symptoms include myopia, night blindness, and photophobia [224].

### 6.2. Cockayne Syndrome (OMIM: 216400)

Cockayne syndrome is classified into three types based on the timing and severity of symptoms [225,226].

Type 1 is progressive, with symptoms such as growth and developmental delays typically appearing after the first year of life, within the first two years. This form is caused by a defect in the *ERCC8* gene.

In Type 2, symptoms are apparent at birth. The causative genes are *CSA* or *CSB* on chromosome 5 (*ERCC6* or *ERCC8*). These genes code for proteins involved in transcriptional processes and DNA repair. The inheritance pattern is autosomal recessive. Systemic signs include short stature, microcephaly, premature aging, and dwarfism. Systemic symptoms include neurological abnormalities, intellectual disability, hearing difficulties, and progeria [227,228,229]. Ophthalmological signs include photophobia and retinopathy with fine granular spots [230].

Type 3 presents later in childhood and is generally a milder form of the disease.

### 6.3. Rud’s Syndrome (OMIM: 308200)

This condition is a rare genodermatosis [231,232,233]. Although it is a genetic disorder, there is no information available regarding the specific gene defect. The inheritance pattern is X-linked recessive.

Systemic signs include congenital ichthyosis, short stature, dwarfism, and hypogonadism. Systemic symptoms include intellectual disability, epilepsy, and polyneuropathy.

Ophthalmological signs include retinitis pigmentosa, strabismus, glaucoma, cataracts, and nystagmus [231].

### 6.4. Alport Syndrome (OMIM: 301050)

Alport syndrome is a heterogeneous disease [234] caused by mutations in the *COL4A5*, *COL4A3*, and *COL4A4* genes. The condition is primarily inherited in an X-linked recessive manner, but autosomal recessive and autosomal dominant forms also exist.

Systemic signs include progressive nephritis, glomerulonephritis, hematuria, and proteinuria. Systemic symptoms include hearing disturbances that can lead to deafness [235,236].

Ophthalmological signs include corneal erosions, keratoconus, lenticonus, and cataracts. Retinal abnormalities may include patchy retinal degeneration with yellow flecks in the macular and mid-peripheral regions of the retina, incomplete foveal hypoplasia, staircase foveopathy, or complete retinitis pigmentosa [236,237,238].

### 6.5. Alagille Syndrome (OMIM: 118450)

Also known as Alagille–Watson syndrome or arteriohepatic dysplasia [239], this condition is caused by mutations in the *JAG1* and *NOTCH* genes. The inheritance pattern is autosomal dominant [240,241].

Systemic signs include cirrhosis, jaundice, pruritus, acholic stools, kidney aplasia with deformed proximal tubules or lipidosis, biliary atresia, xanthomas, cardiac insufficiency, characteristic facial bone features, and spina bifida. Systemic symptoms include liver disturbances, which can appear as early as infancy [241,242].

Ophthalmological signs include strabismus, myopia, corneal opacifications, cataracts, degenerative changes in the photoreceptors, melanin spots, and cystic abnormalities at the level of the pigment epithelium and Bruch’s membrane. Degenerative and cystic abnormalities are also observed in the photoreceptor layer [243].

## 7. Conclusions

In this review, we have provided a comprehensive overview of syndromic retinitis pigmentosa, focusing on its genetic causes, inheritance patterns, and clinical manifestations across a range of systemic syndromes. It is clear that retinitis pigmentosa is by no means an isolated entity; rather, it can occur as part of several syndromic conditions, which can significantly complicate both diagnosis and management. Recognizing RP as a component of broader syndromic presentations highlights the need for an integrative diagnostic approach, wherein ophthalmological symptoms such as night blindness, nystagmus, and progressive vision loss may serve as early indicators of an underlying systemic disease.

Furthermore, the diversity of systemic symptoms associated with syndromic RP—from neurological deficits and metabolic dysfunctions to renal and skeletal abnormalities—necessitates a multidisciplinary approach to patient care. By establishing these associations, this review stresses the importance of collaboration among ophthalmologists, geneticists, and other healthcare specialists in identifying and managing syndromic RP effectively.

In addition, the emergence of genetic therapies holds promise for addressing both the ocular and systemic symptoms of these conditions. While current treatments focus on symptom management, advancing genetic interventions may, in the future, offer targeted approaches to mitigate the progression of both RP and its associated systemic effects. However, larger and more rigorous studies are essential to define the safety, efficacy, and clinical protocols for these therapies.

Ultimately, by bringing attention to the genetic and clinical complexities of syndromic RP, we aim to enhance clinicians’ ability to diagnose and manage these disorders more accurately. This comprehensive approach not only supports timely intervention but also aligns with the broader goals of personalized medicine, where understanding the genetic basis of a patient’s condition can guide more effective, individualized care plans.

## Data Availability

No new data were created or analyzed in this study.
